# SIBLING RELATIONSHIPS AND COGNITIVE TRAJECTORIES IN THE WISCONSIN LONGITUDINAL STUDY

**DOI:** 10.1093/geroni/igae098.1359

**Published:** 2024-12-31

**Authors:** Gina Lee, Michal Engelman, Jooyoung Kong

**Affiliations:** University of Wisconsin Madison, Madison, Wisconsin, United States; University of Wisconsin Madison, Madison, Wisconsin, United States; University of Wisconsin Madison, Madison, Wisconsin, United States

## Abstract

Sibling relationships are positively associated with psychological well-being over time. However, no prior studies have examined the impact of sibling relationships in middle and older adulthood on longitudinal patterns in cognitive functioning. Using four waves (spanning 27 years) of sibling data from the Wisconsin Longitudinal Study (N = 19,046), our study explores the relationship between sibling closeness, emotional support, and contact frequency in 1993, 2004, and 2011 (when participants were roughly 54, 65, and 72) and trajectories in cognitive measures including immediate and delayed recall, digit ordering, and letter fluency in later life (2004, 2011, and 2020). In growth curve models, sibling closeness (measured in 2004) and emotional support received from siblings (measured in 1993, 2004, 2011) were positively associated with baseline levels of letter fluency but not change over time. Next, we employed the sibling dyad data with at least one letter fluency data available from each source (n = 3,243 pairs of siblings) to fit a parallel process model to examine whether siblings’ letter fluency scores moved together over time. We found that both the intercepts and slopes of letter fluency trajectories were positively associated for siblings, implying that participants who experienced a steep decline in letter fluency were likely to have siblings who experienced similarly steep declines. Future studies can further explore what aspects of siblings relationship or shared exposures (e.g. shared family characteristics or environments, spending time together, having similar health behaviors, or providing care for each other) influence the shared cognitive patterns.

